# Myeloid-derived suppressor cells in cancer: therapeutic targets to overcome tumor immune evasion

**DOI:** 10.1186/s40164-024-00505-7

**Published:** 2024-04-12

**Authors:** Junli Lu, Yiming Luo, Dean Rao, Tiantian Wang, Zhen Lei, Xiaoping Chen, Bixiang Zhang, Yiwei Li, Bifeng Liu, Limin Xia, Wenjie Huang

**Affiliations:** 1 Hepatic Surgery Centre, Tongji Hospital, Tongji Medical College, Huazhong University of Science and Technology, Hubei Key Laboratory of Hepato-Pancreato-Biliary Diseases, Wuhan, 430030 Hubei China; 2 Clinical Medicine Research Center for Hepatic Surgery of Hubei Province, Key Laboratory of Organ Transplantation, Ministry of Education and Ministry of Public Health, Wuhan, 430030 Hubei China; 3 The Key Laboratory for Biomedical Photonics of MOE at Wuhan National Laboratory for Optoelectronics-Hubei Bioinformatics and Molecular Imaging Key Laboratory, Systems Biology Theme, Department of Biomedical Engineering, College of Life Science and Technology, Huazhong University of Science and Technology, Wuhan, 430074 China; 4 Department of Gastroenterology, Institute of Liver and Gastrointestinal Diseases, Hubei Key Laboratory of Hepato-Pancreato-Biliary Diseases, Tongji Hospital of Tongji Medical College, Huazhong University of Science and Technology, Wuhan, 430030 Hubei China

**Keywords:** Myeloid-derived suppressor cells, Immune escape, Targeting therapy, Tumor immunology

## Abstract

Paradoxically, tumor development and progression can be inhibited and promoted by the immune system. After three stages of immune editing, namely, elimination, homeostasis and escape, tumor cells are no longer restricted by immune surveillance and thus develop into clinical tumors. The mechanisms of immune escape include abnormalities in antitumor-associated immune cells, selection for immune resistance to tumor cells, impaired transport of T cells, and the formation of an immunosuppressive tumor microenvironment. A population of distinct immature myeloid cells, myeloid-derived suppressor cells (MDSCs), mediate immune escape primarily by exerting immunosuppressive effects and participating in the constitution of an immunosuppressive microtumor environment. Clinical trials have found that the levels of MDSCs in the peripheral blood of cancer patients are strongly correlated with tumor stage, metastasis and prognosis. Moreover, animal experiments have confirmed that elimination of MDSCs inhibits tumor growth and metastasis to some extent. Therefore, MDSCs may become the target of immunotherapy for many cancers, and eliminating MDSCs can help improve the response rate to cancer treatment and patient survival. However, a clear definition of MDSCs and the specific mechanism involved in immune escape are lacking. In this paper, we review the role of the MDSCs population in tumor development and the mechanisms involved in immune escape in different tumor contexts. In addition, we discuss the use of these cells as targets for tumor immunotherapy. This review not only contributes to a systematic and comprehensive understanding of the essential role of MDSCs in immune system reactions against tumors but also provides information to guide the development of cancer therapies targeting MDSCs.

## Introduction

The population of myeloid cells is highly diverse. Myeloid cells include mononuclear phagocytes (MNPs) (encompassing macrophages, monocytes, and dendritic cells [DCs]) and granulocytes (mast cells, neutrophils, eosinophils, and basophils), which play a variety of different and specific roles in protecting the body in response to pathogenic stimuli. However, sustained stimulation by inflammation, chronic infection, or cancer (which involves relatively low-intensity signals) causes sustained myelopoiesis. Although the exact nature of these myeloid cells depends on the pathogenic stimulus in the host, they share several similar features: lack or reduced expression of mature myeloid cell markers, inability to differentiate into mature myeloid cells in the presence of tumor-derived factors, expression of Gr-1 and CD11b molecules in mice, high levels of reactive oxygen species, and activation of arginase I and other molecules. This endows these myeloid cells potential to suppress immune effects both in vitro and in vivo [1]. Reports on immunosuppressive myeloid cells were initially published sporadically beginning in the 1970s and 1980s, based on the fact that co-culture of activated T cells with bone marrow cells suppressed T-cell function [2]. At the beginning of the twentieth century, these myeloid cells were renamed immature cells (ImCs) or myeloid suppressor cells (MSCs). In 2007, the name MDSCs was proposed to unify the descriptions of these cell types [1]. The name is based on the fact that the cells originate from the myeloid lineage and are characterized mainly by their immunosuppressive activity. MDSCs have since been used as a catch-all term in a variety of settings, particularly in the field of cancer biology.

There are two primary types of MDSCs called polymorphonuclear-MDSCs (PMN-MDSCs) and monocytic-MDSCs (M-MDSCs). These cells resemble neutrophils and monocytes phenotypically and morphologically and thus, phenotype and morphology alone are not enough to identify MDSCs. Besides the two main types of cells, MDSCs include a small population of cells (less than 3%) with myeloid colony-forming activity [3]. Murine MDSCs were initially defined as those that expressed the Gr1 and CD11b surface molecules. Therefore, in mice, PMN-MDSCs were defined as CD11b^+^Ly6C^−^Ly6G^+^, and M-MDSCs were defined as CD11b^+^Ly6C^+^Ly6G^−^ [3]. In humans, PMN-MDSCs and M-MDSCs in human peripheral blood can be separated by density gradient centrifugation. Human PMN-MDSCs are often described as HLADR^−^CD11b^+^ CD14^−^ CD15^+^ CD33^Mid^ cells, and M-MDSCs are described as HLADR^−^CD11b^+^ CD14^+^CD15^−^CD33^high^ cells [4].

MDSCs mediate immune escape mainly by exerting immunosuppressive functions. Although MDSCs are involved in suppressing various immune cells, their primary target is T cells. In contrast depletion of MDSCs using specific antibodies enhances T cell infiltration, survival and cytotoxic efficacy driven by bispecific antibody or chimeric antigen receptor [5]. MDSCs exert immunosuppressive effects mainly by generating active ingredients such as arginase 1 (ARG1) [6], reactive oxygen species [7], and nitric oxide [8]. In the tumor microenvironment, MDSCs can display effective immunosuppressive and immune escape effects through a variety of mechanisms: depletion of metabolites essential for T-cell function, production of nitrogen species and reactive oxygen, blockade of lymphocyte homing, expression of ectoenzymes regulating adenosine metabolism, induction of immunosuppressive cells, and expression of negative immune checkpoint molecules [9]. In addition to their effects on immune responses, MDSCs promote tumor development by secreting vascular endothelial growth factor (VEGF) [10] and matrix metalloproteinase-9 (MMP9) [11] to support tumor angiogenesis and expressing CXCR2 to promote the formation of pre-metastatic niche [12].

In the last two decades of research, the immune system has been shown to paradoxically inhibit and support tumor development. This process is known as cancer immunoediting and undergoes 3 main phases, namely, elimination, equilibrium and escape [13]. During the elimination phase, the innate immune system and the adaptive immune system team up to recognize and eliminate cells that have become transformed, evading tumor suppression mechanisms. The few surviving tumor subclones can enter the equilibrium phase, where tumor growth is limited and even stagnates over time. However, subclones of tumors with low immunogenicity can be selected by the adaptive immune system in combination with the genetic instability of tumor cells to evade immune surveillance [14]. This selection process may involve various types of immune modifications rather than the death of tumor subclones. These changes include the selection of tumor variants that are resistant to immune effectors (sometimes referred to as "immunoediting") and the progressive establishment of an immunosuppressive environment within the tumor. These modified tumor cells can then enter the escape phase, and their growth will no longer be restricted by immune surveillance, leading to the development of clinically detectable tumors. The mechanisms of immune escape include abnormalities in antitumor-associated immune cells, the selection of immune resistance to tumor cells, impaired transport of T cells, and the formation of an immunosuppressive tumor microenvironment [15]. All of these processes are involved in different stages of cancer immunoediting. The complexity of the composition and spatial structure of the tumor immune microenvironment has led to the involvement of MDSCs in cancer immunoediting by various forms [16].

In recent years, research has revealed the clinical significance of MDSCs. Various studies have documented the proliferation of MDSCs in several types of human tumors, such as cutaneous melanoma [17], hepatocellular carcinoma [18], breast cancer [19], prostate cancer [20] and lung cancer [21]. In addition, a number of studies have shown that MDSCs are important prognostic biomarkers for cancer development and potential targets for anticancer therapy [22]. MDSCs can suppress the immune response and protect tumor cells from attack by the host immune system, resulting in tumor immune evasion. Targeting MDSCs to activate tumor immunity and reverse immune escape may be a viable option in tumor patients.

In this review, we discuss the biological role of MDSCs in tumor immune escape. In addition, we also review the specific mechanisms by which MDSCs are involved in tumor immune escape in various types of tumors and discuss in detail the approaches used to target MDSCs for cancer treatment.

## Differentiation and accumulation of MDSCs

**Fig. 1:**
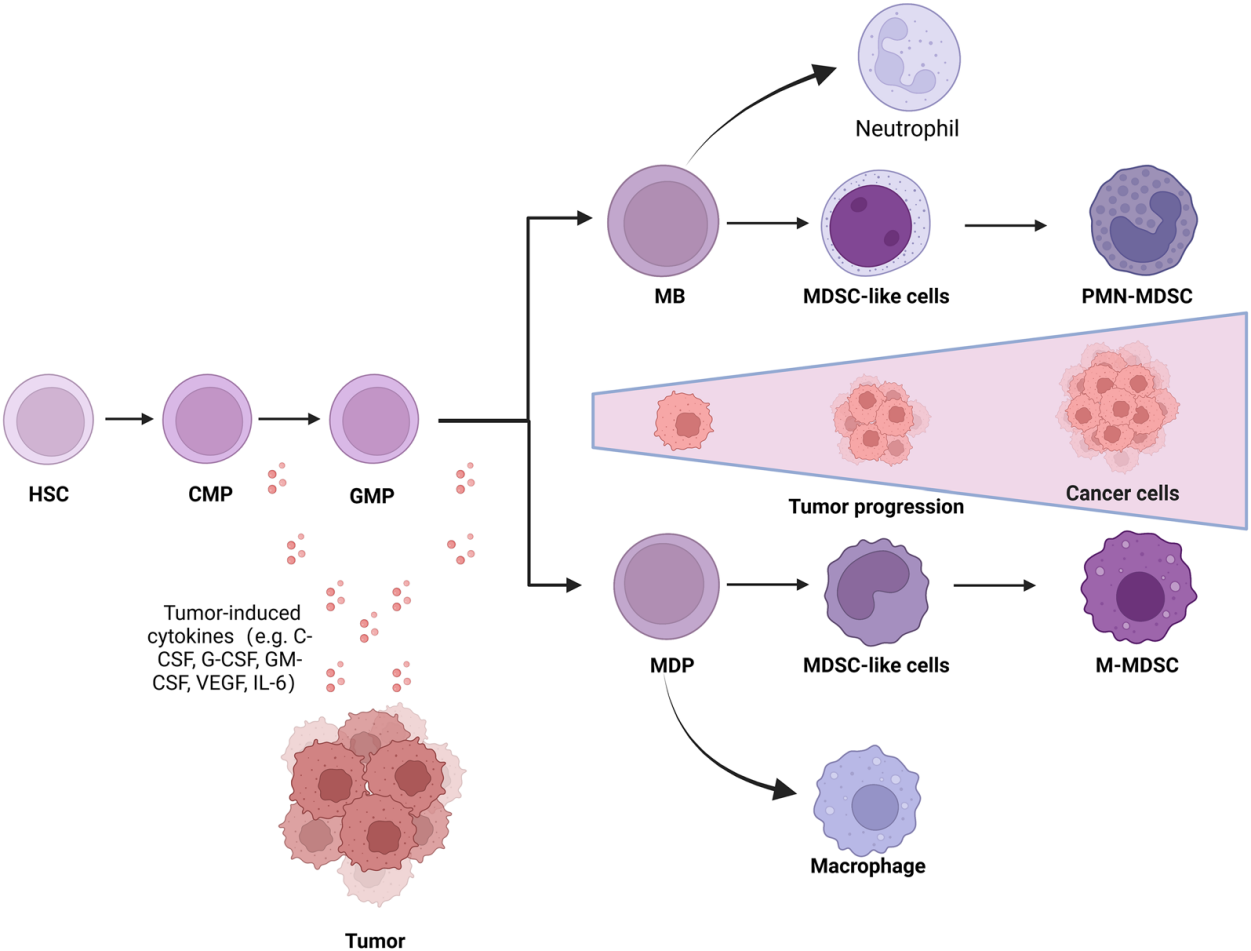
Differentiation and accumulation of MDSCs. MDSCs are differentiated in the bone marrow from hematopoietic stem cells (HSCs) through common myeloid progenitors (CMPs) and granulocyte–macrophage progenitors (GMPs). In response to multiple tumor-induced cytokines, GMPs differentiate to form myeloblasts (MBs), monocytes/macrophages and dendritic cells (MDPs). Most MBs and MDPs can further differentiate into neutrophils and monocytes. However, under pathological conditions, immature myeloid cells expand and transform into immunosuppressive MDSCs. In cancer patients, neutrophils and monocytes, as well as pathologically activated MDSCs, coexist at any given time and accumulate more MDSCs during tumor progression

MDSCs originate from hematopoietic stem cells, common myeloid progenitors (CMPs) and granulocyte–macrophage progenitors (GMPs) [19]. GMPs then differentiate into myeloblasts (MBs), monocytes/macrophages and dendritic cells (MDPs) in reaction to multiple tumor-induced growth signals, cytokines, and other factors [23]. In the early stages of development, these cells with certain biochemical characteristics of MDSCs do not exhibit immunosuppressive activity and can be referred to as MDSCs-like cells. Under continuous stimulation by tumor-secreted factors, MDSCs-like cells expand and transform into immunosuppressive PMN-MDSCs and M-MDSCs (Fig. 1).

In cancer patients, neutrophils, monocytes and pathologically activated MDSCs coexist at any stage. As the tumor progresses, MDSCs further accumulate in the tumor immune microenvironment (Fig. 1). The accumulation of MDSCs is a complex phenomenon whose process can be described by a model that requires two different but partially overlapping signal types. The first one is responsible for the proliferation of immature myeloid cells in connection with the suppression of their terminal differentiation. The second one is responsible for the pathological activation of these cells and the transformation of immature myeloid cells into MDSCs [24]. The development of MDSCs in the tumor context can be divided into 4 main steps. First, factors such as Interleukin-17A (IL-17A), granulocyte–macrophage colony-stimulating factor (GM-CSF), granulocyte colony-stimulating factor (G-CSF) and tumor necrosis factor-alpha (TNF-α) from the tumor site enter the blood and then stimulate bone marrow production. Next, directed by several key chemokine receptors, such as C–C chemokine receptor 2 (CCR2) and C–C chemokine receptor 5 (CCR5), myeloid cells rapidly proliferate during myelopoiesis from the bone marrow and possibly secondary lymphoid organs into the blood. Then, under the action of chemokines, MDSCs home to the tumor site and accumulate. The final step is retention at the tumor site [25].

## The functions of MDSCs

**Fig. 2:**
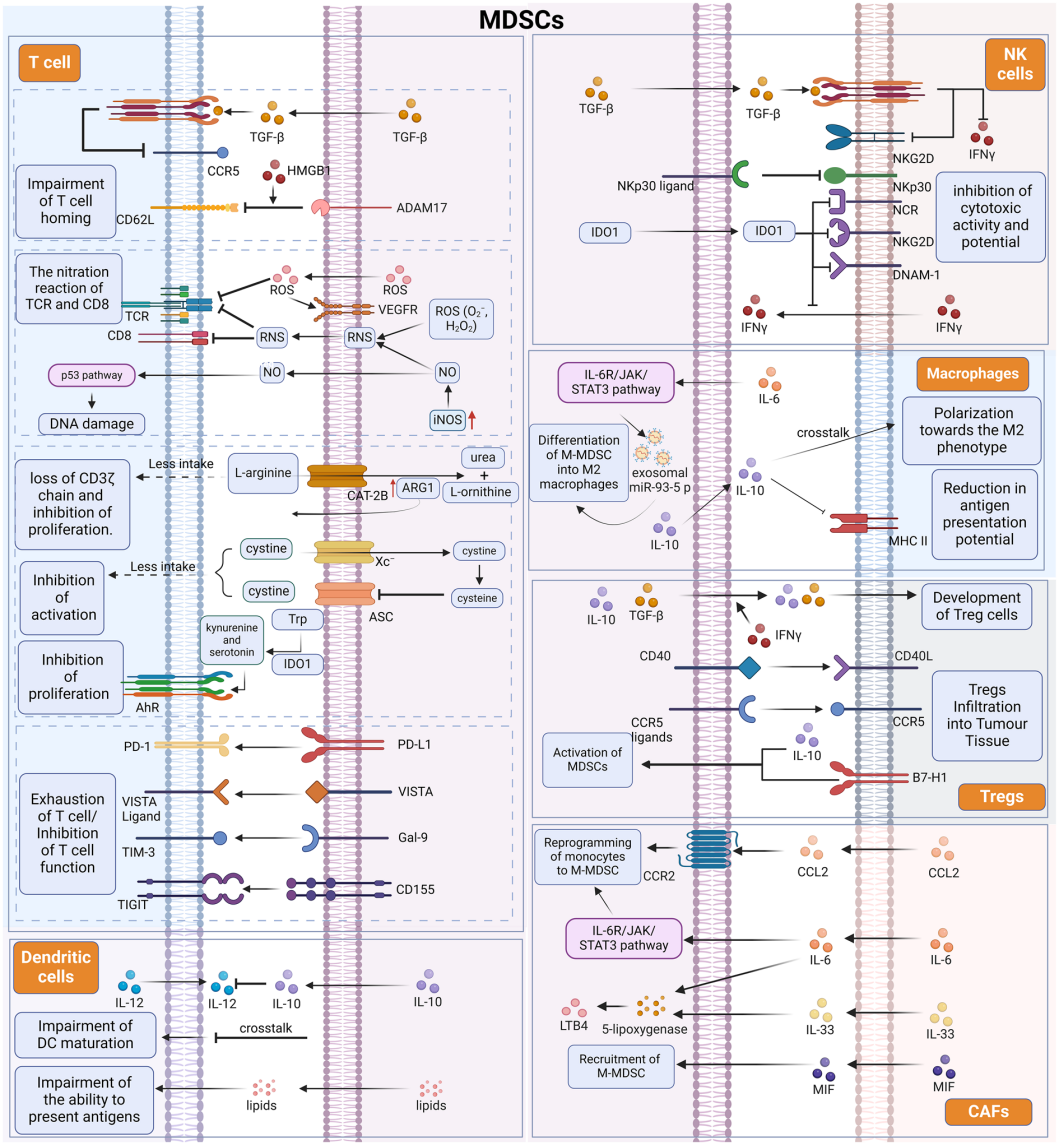
MDSCs interactions with immune cells. MDSCs inhibit T-cell activity through several mechanisms, including impairment of T-cell homing, generation of oxidative stress, depletion of amino acids needed for T-cell responses, and expression of negative immune checkpoint molecules. In addition, MDSCs crosstalk with other immune cells to exert immunosuppressive effects. These include tumor-killing immune cells, such as natural killer (NK) cells and DCs, and immunosuppressive macrophages and regulatory T cells (Tregs). As an important component of the tumor microenvironment, the interaction between MDSCs and CAFs is also critical for tumor development

Immunosuppression is a major function and feature of MDSCs, which enables MDSCs to be differentiated from neutrophils and monocytes in the peripheral blood of human and mouse spleens. Over the past few years, extensive evidence has shown the remarkable characterization and biological role of MDSCs in obesity, pregnancy, infectious diseases and autoimmunity [26]. In cancer, MDSCs function primarily to inhibit antitumor immunity and promote tumor immune escape through multiple mechanisms. This effect is mainly achieved through the interaction of MDSCs with multiple immune cells within the tumor microenvironment (Fig. 2).

## Blockage of T-cell homing

MDSCs can interfere with T-cell immunity by preventing naive T cells from homing to lymph nodes in which they could become activated. Through transforming growth factor beta (TGF-β) signaling, MDSCs disrupt HBV‐specific T-cell trafficking by downregulating CCR5 on them [27]. In addition, MDSCs have been shown to mediate the downregulation of the cell adhesion molecule L-selectin2 (CD62L) on T cells through the expression of the surface metalloprotease a disintegrin and metalloproteinase domain 17 (ADAM17), also called TNF-α-converting enzyme (TACE) [28]. It has been shown that tumor-expressed high mobility group box-1 (HMGB1) also enhances MDSCs-mediated downregulation of L-selectin on naive T cells [29]. This results in decreased homing and antigen-driven activation of lymph node CD8 + T cells [30].

## Inhibition of T-cell function through oxidative stress

MDSCs can induce immunosuppression by generating reactive oxygen species (ROS), which results in an oxidative stress response, promoting MDSCs expansion and suppressing the immune response of T cells [31]. Increased ROS levels also stimulate elevated expression of VEGF receptors on MDSCs, which facilitates MDSCs recruitment into the tumor microenvironment [32]. Therefore the combination of anti-VEGF and PD-1 blockade may exert better anti-tumor immune efficacy [33, 34]. In addition, ROS can catalyze the nitration reaction of T-cell receptor (TCR)/CD8 molecules and prevent TCR/major histocompatibility complex (MHC)-peptide interactions [35]. MDSCs also overexpress inducible nitric oxide synthase and produce large quantities of reactive nitrogen species (RNS), mainly nitric oxide (NO) [36]. Rapid binding of O2^−^ to NO to form RNS can lead to nitration or nitrosylation of TCR/CD8 proteins, ultimately resulting in impaired recognition of TCR/MHC-peptide [37]. NO also drives P53 pathway activation in T cells to cause DNA damage, resulting in severe impairment of T-cell proliferation and survival [38].

## Consumption of amino acids needed for T-cell function

MDSCs can impair T-cell function by reducing metabolites and factors critical to the immune system, such as L-arginine, cysteine and tryptophan (Trp) [39]. A variety of tumor microenvironment (TME)-derived factors induce the upregulation of cationic amino acid transporter protein (CAT-2B) and ARG1 expression in MDSCs. CAT-2B was able to transfer extracellular L-arginine into MDSCs, followed by degradation of L-arginine to urea and L-ornithine catalyzed by ARG1 [40]. In tumor patients, MDSCs have been found to deliver ARG1 to the extracellular environment to promote extracellular L-arginine depletion [41]. Thus, a reduction in the extracellular space arginine concentration can result in the loss of the CD3ζ chain and significant inhibition of T-cell proliferation [42]. Furthermore, MDSCs can take up cystine and metabolize it to cysteine via the xc- transporter, but because of the absence of the neutral amino acid transporter, MDSCs are unable to transport cysteine back to the extracellular level, thus affecting T-cell activation [43]. Furthermore, MDSCs also reduce Trp levels through indoleamine-2,3-dioxygenase 1 (IDO1) expression in external environment, thereby preventing T-cell development through the general control non-repressed 2 pathway [44, 45]. The production of kynurenine and serotonin due to Trp depletion activates the aryl hydrocarbon receptor (AhR) to trigger IDO1 production and an anti-inflammatory reaction [45].

## Expression of negative immune checkpoint molecules on MDSCs

PD-L1 is a key negative regulator of the immune system that mediates immune escape in tumors [46], and PD-L1 expression on MDSCs is closely associated with immunosuppression. Consistent with the immunosuppressive activity of MDSCs, it has been proven that blocking MDSCs improves the antitumor effect of programmed cell death 1 (PD-1) inhibitors in mice, which can be in conjunction with enhanced CD8 + T-cell infiltration in tumors and reduced expression of immunosuppressive proteins such as arginase 1, S100A8, S100A9, and iNOS by MDSCs [47]. In addition, several other immune checkpoint molecules, including V-domain Ig suppressor of T-cell activation (VISTA), galactose lectin-9 (Gal-9) and CD155, are involved in immune suppression mediated by MDSCs. High VISTA expression on MDSCs in the peripheral blood of patients with acute myeloid leukemia (AML) strongly correlates with PD-1 expression on T cells [48]. VISTA expression is enhanced on tumor-infiltrating MDSCs and linked to areas of severe hypoxia in the TME, and antibodies targeting or genetically ablating VISTA under hypoxia alleviate MDSCs-induced T-cell suppression [49]. Gal-9 on MDSCs can interact with T-cell immunoglobulin and mucin structural domain 3 (TIM-3) expression on T cells to expand MDSCs and suppress T-cell reactions [50]. Moreover, Gal-9 from nasopharyngeal carcinoma cells upregulated the expression of several pro-inflammatory cytokines essential to MDSCs differentiation, including IL-1β and IL-6. The process is based on enhanced interferon gene (STING) protein catabolism resulting from direct interaction of Gal-9 carbohydrate recognition domain 1 with the STING C-terminus and subsequent enhancement of K48-linked ubiquitination of STING via the E3 ubiquitin ligase tripartite motif‐containing (TRIM) 29 [51]. T-cell immunoglobulin and the ITIM domain (TIGIT) is a suppressive regulatory factor that has been shown to have an immunosuppressive effect on antitumor immunity in a wide range of solid tumors and leukemias [52]. In head and neck squamous cell carcinoma (HNSCC), CD155 expression on MDSCs promoted MDSCs-mediated T-cell suppression, and in vitro blocking the TIGIT/CD155 pathway with anti-TIGIT antibodies substantially inhibited MDSCs immunosuppressive capacity and enhanced the antitumor immune response [53].

## Crosstalk between MDSCs and other immune cells involved in the TME

Although MDSCs primarily target effector T cells, recent reports have proven that MDSCs can also mediate immune escape through the inhibition of other tumor-killing immune cells, such as DCs and natural killer (NK) cells. DCs are the other major myeloid cells infiltrating into the TME. Although signals from the TME promote the influx of immature DCs, multiple factors, including adenosine accumulation, lactate accumulation, and hypoxic conditions, induce DC dysfunction [54]. In addition, it has been suggested that crosstalk between DCs and MDSCs may also be partly responsible for the decreased DC function. When bone marrow-derived MDSCs are co-cultured with DCs in vitro, the DC population decreases as the number of MDSCs increases [55, 56]. Studies of MDSCs in melanoma patients have shown that high frequencies of M-MDSCs impair DC maturation by reducing antigen uptake, preventing migration of immature and mature DCs, skewing DC cytokine production toward an anti-inflammatory phenotype, and blocking the ability of DCs to induce IFNγ-producing T cells [57]. It was also found in a mouse model that increased interleukin-10 (IL-10) production by MDSCs in hepatocellular carcinoma inhibited the secretion of interleukin-12 (IL-12) by DCs [58]. In addition, PMN-MDSCs produce oxidatively truncated lipids that can be transferred to DCs, attenuating the ability of DCs to cross-present antigens [59].

One of the main mechanisms of MDSCs-induced NK cell incompetence is the reduction in natural killer group 2D (NKG2D) and interferon-γ (IFN-γ) expression in NK cells via TGF-β, which thereby inhibits cytotoxic potential under tumor conditions [60]. When MDSCs are adoptively transferred to tumor-bearing mice, the cytotoxic activity of NK cells can be inhibited by reducing the levels of perforin in NK cells [61]. Furthermore, MDSCs can also inhibit NK cell function through the NKp30 receptor or by downregulating the expression of CD247 on NK cells [62, 63]. In addition, MDSCs-expressed IDO reduces NK cell activity by downregulating receptors such as NCR, NKG2D and DNAM-1 and decreasing IFN-γ secretion from NK cells [64, 65]. This inhibition can be regulated by blocking signal transducer and activator of transcription 3 (STAT3)-mediated nuclear factor-κB (NF-κB) activation [66].

In addition to their ability to suppress immune T cells to destroy tumors, MDSCs may also be involved in tumor immune escape by stimulating other immune suppressor cells, such as macrophages and regulatory T (Treg) cells [67]. MDSCs not only are a source of tumor-associated macrophages but also may influence macrophage activation status, function, and polarization through association [68]. Driven by IL-6, the IL-6R/JAK/STAT3 pathway is activated in PMN-MDSCs, which in turn causes the synthesis and secretion of exosomal miR-93-5p, driving differentiation of M-MDSCs into M2 macrophages [69]. In the tumor microenvironment, MDSCs crosstalk with macrophages mainly through the production of IL-10, which promotes macrophage polarization toward the M2 phenotype. IL-10 produced by MDSCs also biases the differentiation of the helper T-cell population toward the Th2 phenotype, which in turn affects the development of cytotoxic T lymphocytes. Th2 cells also produce high levels of IL-4, which in turn promotes TAM development [70]. In addition, IL-10 production by MDSCs could also decrease the antigen-presenting potential of macrophages by affecting MHC II expression [71]. In a mouse model of colon cancer, increased secretion of IL10 and TGF-β by MDSCs after IFN-γ stimulation promoted the development of CD4 + CD25 + Treg cells [72]. In an A20 B-cell lymphoma model, MDSCs overexpressing low levels of MHC II have been reported to act as tolerogenic antigen-presenting cells (APCs) capable of antigenic uptake and presentation to tumor-specific Treg cells in an arginase-induced manner [73]. When adoptive transfer of MDSCs was performed from CD40-deficient mice, it failed to induce expansion and tolerance of tumor-specific Treg cells. This finding suggests, in part, a role for CD40/CD40L interactions in the crosstalk between MDSCs and Treg cells [74]. In mouse models of cancer, M-MDSCs inside tumors can generate CCR5 ligands to attract Tregs with high levels of CCR5 to infiltrate tumor tissue [75]. Additionally, in a mouse model of melanoma, Treg cells promoted MDSCs function by enhancing the levels of B7 family members of immunomodulatory ligands, such as B7-H1 (also known as PD-L1), B7-H3, and B7-H4, as well as the generation of IL-10 in MDSCs [76].

TME is a highly complex system. In addition to tumor cells and infiltrating immune cells, cancer-associated fibroblasts (CAFs) are also an important part of it. Under the influence of various cytokines and chemokines released by CAFs, MDSCs infiltrate and generate inside the tumor, thus inhibiting the anti-tumor activity of effector T cells. It has been reported that MDSCs may migrate to tumor sites induced by CAFs-activated STAT3-CCL2 signaling [77]. For example, in lung squamous cell carcinoma, CCR2 + monocytes are induced to migrate toward the tumor site by CAFs-secreted CCL2 and are then reprogrammed to M-MDSCs [78]. Another study described a similar role for CAFs-secreted CCL2 in recurrent bladder cancer [79]. In addition, STAT3 signaling was activated in recruited monocytes in hepatocellular carcinoma induced by IL-6 secreted by CAFs and promoted monocyte differentiation into M-MDSCs [80]. CAFs within hepatocellular carcinoma can also recruit M-MDSCs to hepatocellular carcinoma tissues by promoting macrophage migration inhibitory factor (MIF) secretion in a CD36-dependent manner [81]. The importance of CAFs-secreted IL-6 in the differentiation of MDSCs was reconfirmed in a study of esophageal squamous cell carcinomas, where it was observed that CAFs-derived exosome-packed microRNA-21 (miR-21) also generates M-MDSCs by activating STAT3 signaling [82]. Furthermore, IL-6 and IL-33, which are mainly expressed by CAFs, mediated the metabolism of the over-activated 5-lipoxygenase in MDSCs, and promoted the synthesis of leukotriene B4 (LTB4) in MDSCs to enhance the stemness of intrahepatic cholangiocarcinoma [83].

## The role of MDSCs in common tumors

### Cutaneous melanoma

**Fig. 3:**
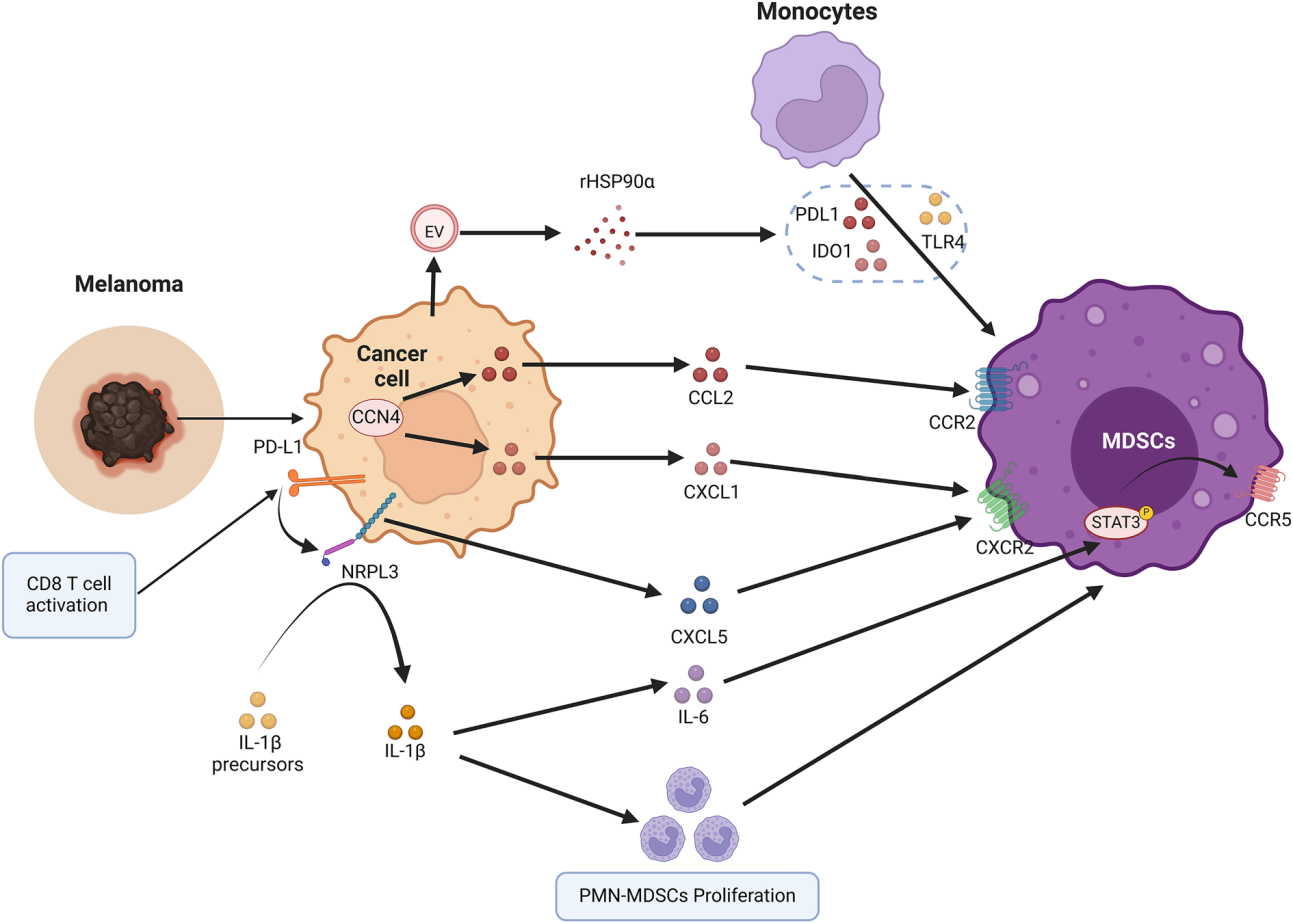
MDSCs in cutaneous melanoma. In melanoma, MDSCs differentiate from monocytes through the action of HSP90α on EVs secreted by tumor cells. In addition, MDSCs are stimulated by secreted IL-1β, IL-6, CCL2, CXCL1 and CXCL5 or induced by tumor cells; proliferate, activate and are recruited to the tumor site, where they in turn exert immunosuppressive effects

Cutaneous melanoma, the most common skin cancer, has increased in incidence in recent decades. Although melanoma can be treated with surgery at early diagnosis, the mortality rate of melanoma remains high due to its aggressive nature, rapid metastatic development and treatment resistance [84, 85]. Like many tumors, melanoma acquires various immunosuppressive mechanisms mediated by immunomodulatory cells, including MDSCs, which synergize with each other to promote immune escape (Fig. 3). High expression of MDSCs infiltration in melanoma patients are associated with tumor stage, metastasis and poor outcome [86]. In patients with unresectable melanoma, high expression of MDSCs is negatively associated with clinical response to ipilimumab [17] and may predict the failure of anti-PD-1 s-line immunotherapy [87].

It has been reported that bone marrow cells are transformed into MDSCs under the influence of tumor-secreted extracellular vesicles (EVs) [88]. In melanoma, M-MDSCs are generated from monocytes stimulated by TLR4 signaling induced by heat shock protein 90α (HSP90α) on EVs that stimulate PD-L1 and IDO1 expression [89]. Activation of NOD-like receptor protein 3 (NLRP3) and formation of the NLRP3 inflammasome in melanoma have been reported [90]. Upon NLRP3 activation, inactive IL-1β precursors are processed to mature IL-1β by cystathionin-1β [91]. Following the activation of NLRP3 in melanoma cells, PMN-MDSCs proliferate in response to IL-1β-induced melanoma-associated inflammation, which results in reduced natural killer and CD8 + T cell activity and enhanced Treg cell population in primary tumors. Together, these factors lead to immunosuppression [90]. In addition, it has been found that the level of immunosuppressive genes in PMN-MDSCs increases in response to stimulation by the IL-6/STAT3 signaling pathway, which is driven by NLRP3-dependent IL-1β production [92]. Moreover, IL-6 upregulates CCR5 expression in PMN-MDSCs, recruited PMN-MDSCs into the TME and led to the increased immunosuppressive capacity of CCR5-related PMN-MDSCs [93]. Therefore, targeting NLRP3 in tumors to inhibit the immunosuppressive function of MDSCs may constitute an investigational strategy for the treatment of melanoma, especially in the environment of immunotherapy-resistant tumors.

After their accumulation and activation in the bone marrow, MDSCs are attracted to the tumor site by a group of chemokines. Although CCL2, CCL3, and CCL4 are important for the recruitment of M-MDSCs through CCR2 [94], the ligands CXCR2, CXCL1, CXCL2, CXCL3, CXCL5, CXCL6, and CXCL7 mainly mediate PMN-MDSCs migration [95]. PMN-MDSCs infiltration within melanoma tissues is a significant contributor to primary melanoma growth and metastasis. PMN-MDSCs were shown to infiltrate primary melanoma and metastases via CXCL1/CXCR2 interactions [96]. In mice with melanoma, PMN-MDSCs produce hepatocyte growth factor and TGF-β, stimulating epithelial-to-mesenchymal transition and tumor spread [97]. Cell Communication Network Factor 4 (CCN4) is a secretory stromal cell protein generated by activation of the Wnt/β-catenin pathway that promotes the metastatic spread of melanoma by participating in the epithelial-mesenchymal transition [98]. In addition, when CCN4-knockout melanoma cells were implanted into immunocompetent mice, the infiltration of PMN-MDSCs was reduced. This was because local CNN4 expression inhibited the release of IFN from CD8 + T cells and increased tumor secretion of MDSCs-attracting chemokines such as CCL2 and CXCL1 [99]. In addition, in multiple preclinical tumor models as well as clinical specimens, activation of CD8 T cells in answer to PD-1 blockade triggers a PD-L1/NLRP3 inflammatory signaling cascade that eventually causes PMN-MDSCs recruitment into melanoma tissue, resulting in immune suppression and thus immune escape [100]. PMN-MDSCs infiltration in tumors can be inhibited by NLRP3 blockade, significantly improving the efficacy of anti-PD-1 antibody immunotherapy [100].

### Hepatocellular carcinoma

**Fig. 4:**
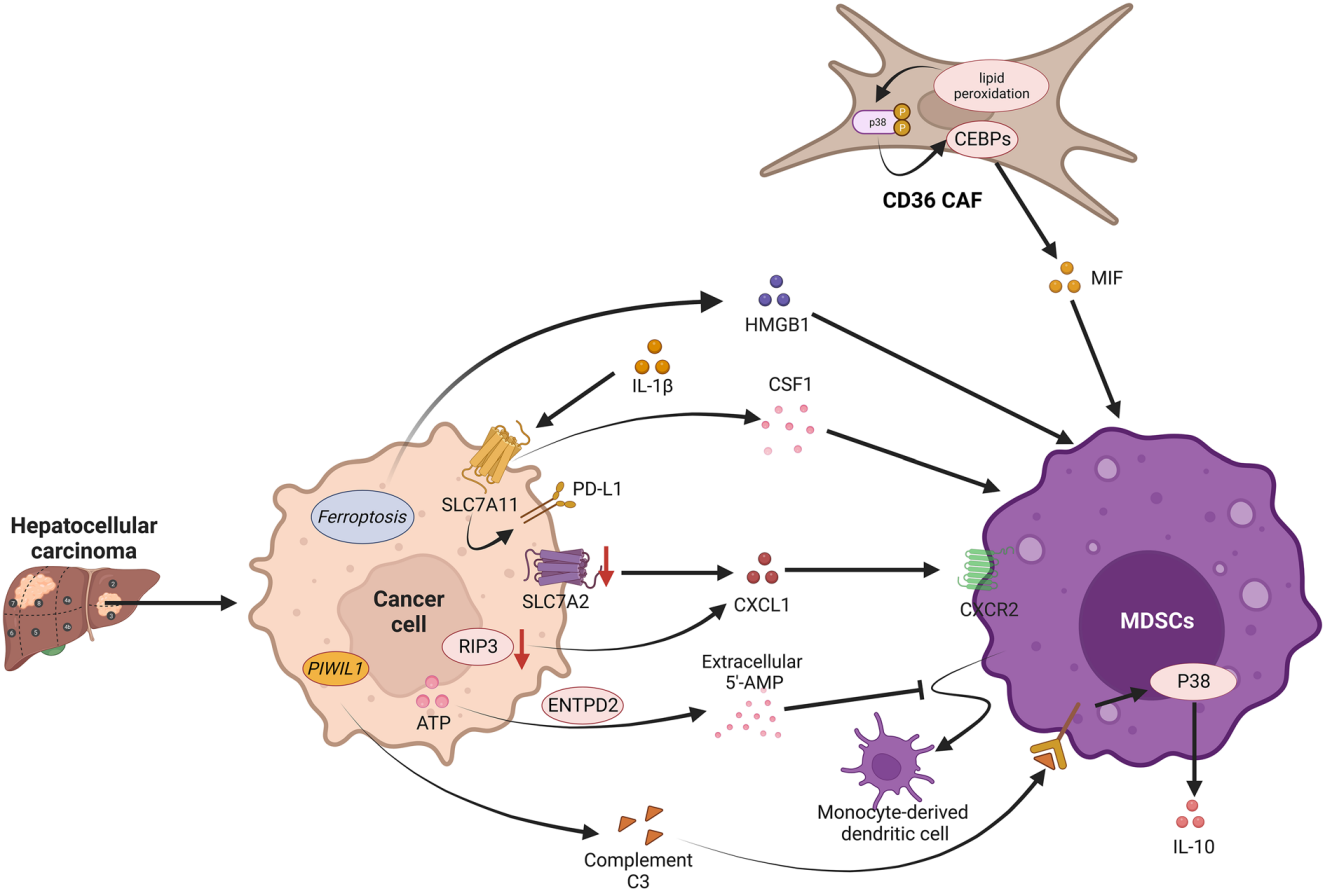
MDSCs in hepatocellular carcinoma. In hepatocellular carcinoma, MDSCs are recruited to HCC cells and enhance immunosuppression, which is stimulated by CAFs secreting MIF and tumors secreting HMGB1, CSF1 and CXCL1. In addition, tumor overexpression of ENTPD2 converts extracellular ATP to 5'-AMP, which maintains MDSCs and prevents MDSCs from differentiating. Under the stimulation of complement C3, the interaction between MDSCs and HCC cells was enhanced, which initiated the expression of IL10 and suppressed T-cell immunity

Hepatocellular carcinoma (HCC) is a leading cause of cancer death worldwide. Inflammation is strongly linked to hepatocellular carcinoma, especially hepatitis and cirrhosis [101]. Clinical studies over the past decade have demonstrated the clinical significance of MDSCs in patients with hepatocellular carcinoma [102–104]. In patients with hepatocellular carcinoma, CD14(+)HLA-DR(low/−) MDSCs were markedly upregulated in the peripheral blood or tumor tissue. MDSCs from HCC were unable to stimulate allogeneic T-cell responses and had high arginase activity [18]. This finding suggested that hepatocellular carcinoma drives MDSCs to infiltrate, recruit and suppress effector T-cell function within the TME through various mechanisms (Fig. 4). Analysis of single-cell sequencing in mouse HCC models and human HCC organoids suggested that this difference may be due to METTL1-mediated accumulation of PMN-MDSCs following insufficient radio frequency treatment, which suppresses antitumor immunity and promotes HCC progression [105].

In the TME, oxidized LDL produced by dyslipidemic metabolism induces activation of the lipid peroxidation/p38 phosphorylation/CEBP axis within CD36 CAFs and ultimately promotes macrophage MIF secretion in a CD36-dependent manner [81]. M-MDSCs stimulated by MIF are recruited to HCC tissues and enhance immunosuppression in the TME [81, 106]. Ferroptosis is an iron-dependent type of cell death that leads to cell membrane destruction through the accumulation of lipid peroxides [107]. In HCC, ferroptosis does not provide cell-autonomous tumor suppression but triggers tumor infiltration of MDSCs via HMGB1, thereby eliciting an adaptive immune response [108]. In addition, inflammatory factors in the TME play essential roles in MDSCs-mediated immune escape in HCC. It has been shown that IL-1β-induced solute carrier family 7 member 11 (SLC7A11) overexpression upregulates PD-L1 and colony-stimulating factor 1 (CSF1) through the α-ketoglutarate/HIF1α axis, promoting MDSCs recruitment and infiltration in the hepatocellular carcinoma tumor niche [109]. Interleukin-33 (IL-33)-treated mice presented an increased incidence of M-MDSCs and a decreased incidence of PMN-MDSCs [110]. Solute carrier family 7 member 2 (SLC7A2), which is also a constituent of the SLC carrier family, was found to be commonly lacking in most patients with hepatocellular carcinoma [111]. MDSCs recruitment is driven by a defect in SLC7A2 within tumors through the upregulation of CXCL1 levels via the phosphatidylinositol 3-kinase (PI3K)/protein kinase B (AKT)/NF-κB axis [111]. By phosphorylating P65^Ser536^and promoting P65^Ser536^ nuclear translocation, receptor-interacting protein kinase 3 (RIP3) deficiency in HCC promotes CXCL1/CXCR2-induced MDSCs chemotaxis [112]. MDSCs may accumulate substantially in hepatocellular carcinoma under the combined effect of the above mechanisms, thus exerting immunosuppressive effects to promote immune escape.

Hypoxia is an important environmental factor in hepatocellular carcinoma [113]. In HCC, hypoxia induces the ectodomain, ectonucleoside triphosphate diphosphohydrolase 2 (ENTPD2), mainly through hypoxia-inducible factor-1 (HIF-1), leading to its overexpression [114]. ENTPD2 converts extracellular adenosine triphosphate (ATP) to 5'-Adenosine monophosphate (5'-AMP), which maintains the state of M-MDSCs and prevents M-MDSCs differentiation [114].

It has been shown that metabolites produced by cancer cells can promote tumor development by modulating the functional phenotype of different immune cells [115, 116]. In hepatocellular carcinoma, Piwi Like RNA-Mediated Gene Silencing 1 (PIWIL1) increases fatty acid metabolism via mitochondrial fatty acid β-oxidation (FAO) to accelerate energy production for rapid tumor growth [117]. PIWIL1-induced FAO activates complement C3 by inducing oxidative stress. Complement C3 facilitates the engagement between HCC cells and PMN-MDSCs through p38 MAPK signaling activated in PMN-MDSCs, which in turn initiates the expression of the immunosuppressive cytokine IL10 to suppress T-cell immunity [117]. It was concluded that androgen (AR)-activated cell cycle-related kinase (CCRK) signaling is the main pathway leading to male predominance in human HCC [118, 119]. Self-reinforcing circuits involving CCRK constitute the vicious epigenetic circuitry in HCC [120]. It has been suggested that the self-reinforcing CCRK circuit may induce immunosuppression of PMN-MDSCs through Enhancer of zeste homolog 2 (EZH2)/NF-κB/IL-6 signaling, which in turn suppresses effector T cells [103].

### Breast cancer

Breast cancer (BC) is the most common cancer diagnosis and the major cause of cancer death in women [121]. Although immunotherapy has not yet become a common therapy for patients with BC, a pooled report of 1954 breast tumors showed that BC can be classified as immune-privileged and immune-privileged according to different degrees of immunogenic sensitivity. These classifications have exhibited noteworthy variations in terms of distant m0etastasis-free survival [122]. During the development of breast cancer, cancer cells secrete a large number of cytokines that affect the differentiation of bone marrow cells and promote MDSCs development [19, 54]. In a mouse model of breast cancer, MDSCs features included several genes related to immunomodulation, such as arginase 2 and Cd84, and chemokine receptors (e.g., Ccr2 and Cxcr2), suggesting that MDSCs can be directed into tumor tissue by chemokines [123]. The combination of CD84 and JAML cell surface receptors on MDSCs with CD11b/Gr1 staining detects the presence of MDSCs in mouse tumor tissues or in humans with CD11b/CD14 or CD15 [123].

PMN-MDSCs differentiate from granulocyte–monocyte progenitors (GMPs) and granulocyte progenitors (GPs) in the bone marrow (BM) through the binding of C–C motif chemokine ligand 20 (CCL20), which is expressed at high levels in BC cells, to its receptor CCR6 [124]. CCL20-regulated PMN-MDSCs secrete large amounts of CXCL1 by binding to CXCR2 and activating the NOTCH1/HEY2 signaling pathway in BC cells, leading to an increase in breast cancer stem cells (BCSCs). MDSCs in breast cancer induce IL-6-dependent STAT3 phosphorylation and NOTCH activation via nitric oxide (NO), resulting in prolonged STAT3 activation, which enhances the stem cell-like properties of breast cancer stem cells and inhibits T-cell activation to promote tumor formation [125]. In addition, MDSCs activate the PI3K/AKT/NF-κB pathway in B cells through the PD-1/PD-L1 axis, inducing the generation of PD-1-negative, PD-L1-positive B cells with immunosuppressive functions to suppress T-cell immune responses [126]. MDSCs may promote the formation of an immunosuppressive TME by suppressing the immune function of T cells through the above mechanism.

To meet their bioenergetic and biosynthetic needs, tumor cells reprogram metabolic pathways, such as preferential aerobic glycolysis, which is considered one of the hallmarks of tumors [127]. In triple-negative BC mouse models, glycolytic metabolism may repress specific CCAAT/enhancer-binding protein beta (CEBPB) isoforms and liver-enriched activator protein (LAP) via AMP-activated protein kinase (AMPK)-ULK1 and autophagic pathways to effectively stimulate tumor G-CSF and GM-CSF expression and to maintain MDSCs development and escape immunity [128]. MDSCs amplify and aggregate under the influence of IL-33-induced autocrine GM-CSF in the breast cancer tumor microenvironment and maintain the survival of MDSCs [129]. Moreover, the activation of NF-κB and mitogen-activated protein kinase (MAPK) signaling in MDSCs combined with IL-33-induced ARG1 expression enhances the immunosuppressive ability of MDSCs [129]. Cancer cell-derived GM-CSF also induces transcription of the genes encoding AMP-activated protein kinase alpha (AMPKα) and Prkaa1 in tumor-MDSCs, regulating the differentiation of M-MDSCs to TAMs and exerting immunosuppressive effects [130].

Furthermore, the development of early myeloid-derived suppressor cells (eMDSCs) in BC was induced by tumor exosome-derived miR-9 and miR-181a, which activated the Janus kinase (JAK)/STAT signaling pathway by targeting suppressor of cytokine signaling-3 (SOCS3) and protein inhibitor of activated STAT-3 (PIAS3), respectively [131]. Additionally, acetylation of Smad family member 3 (SMAD3) at K20 and K117 by lysine acetyltransferase 6A (KAT6A) enhanced SMAD3 binding to the oncogenic chromatin modifier TRIM24 and disrupted the binding of SMAD3 to the tumor suppressor TRIM33 [132]. In turn, this leads to the recruitment of the TRIM24-SMAD3 complex to chromatin through KAT6A acetylation of histone H3 lysine 23, which increases immune-associated cytokine expression and leads to MDSCs recruitment and immune escape in triple-negative breast cancer through immunosuppression [132]. Adenosinergic metabolites produced by high ectonucleotide pyrophosphatase/phosphodiesterase 1 (Enpp1) expression in breast cancer enhance the expression of haptoglobin, which recruits PMN-MDSCs [133]. PMN-MDSCs infiltration causes immunosuppression, allowing Enpp1high circulating tumor cells to promote relapse through a self-seeding mechanism that causes locoregional failure [133].

### Prostate cancer

Prostate cancer (PCa) is the most prevalent male-associated cancer and the second major cause of cancer-related deaths in men [121]. Patients with a wide range of cancer types can achieve durable therapeutic responses with immune checkpoint blockade (ICB). However, castration-resistant prostate cancer (CRPC) shows overwhelming de novo tolerance to ICB [134]. MDSCs, which play an essential role in tumor immune escape, were found to be significantly more frequent and absolute in PCa patients than in healthy individuals [135]. More importantly, the increased frequency of M-MDSCs is linked to known negative prognostic markers in PCa patients, further suggesting that high levels of M-MDSCs are correlated with shorter median overall survival [20]. When ICB was combined with MDSCs-targeted therapy, CRPC exhibited a strong synergistic response [136]. However, the role of MDSCs in the development of PCa and the emergence of CRPC has not been determined.

Interleukin-23 (IL-23), generated by MDSCs, acts as a modulator of pro-tumor immunity and regulates prostate cancer castration resistance by maintaining AR signaling [137]. IL-23 secreted by PMN-MDSCs is a major player in endocrine drug resistance in prostate cancer [137]. Therefore, direct inhibition of PMN-MDSCs can reverse ADT resistance in patients with advanced prostate cancer [137]. Additionally, MDSCs-derived exosomes in the tumor environment promote tumor progression by polarizing macrophages [138]. In PCa, exosome-mediated S100A9 metastasis from MDSCs to PCa cells stimulates PCa cell proliferation, invasion and migration through upregulation of circMID1 (hsa_circ_0007718), which ultimately promotes CRPC progression [139].

In addition, PMN-MDSCs with high levels of STAT3 activity and ARG1 expression are strongly related to prostate cancer progression, and STAT3 blockade impairs the immunosuppressive effect of PMN-MDSCs on effector T-cell activity [140]. In PCa, genetic inactivation of phosphatase and tensin homolog (PTEN) is found in 40% of cases and is associated with poor prognosis and increased metastasis [141]. In PTEN-deficient PCa, chromodomain-helicase-DNA-binding protein 1 (CHD1) is necessary for MDSCs recruitment, which activates the NF-κB network and thereby promotes increased IL-6 secretion [142]. Mast cells, as key stromal accomplices in prostate cancer, can influence the adenocarcinoma and neuroendocrine histotype balance [143, 144]. Direct interaction between PMN-MDSCs and mast cells through CD40L-CD40 binding contributes to enhanced PMN-MDSCs suppressive activity, which in turn inhibits the antitumor response of effector T cells to promote prostate cancer [145].

### Lung cancer

**Fig. 5:**
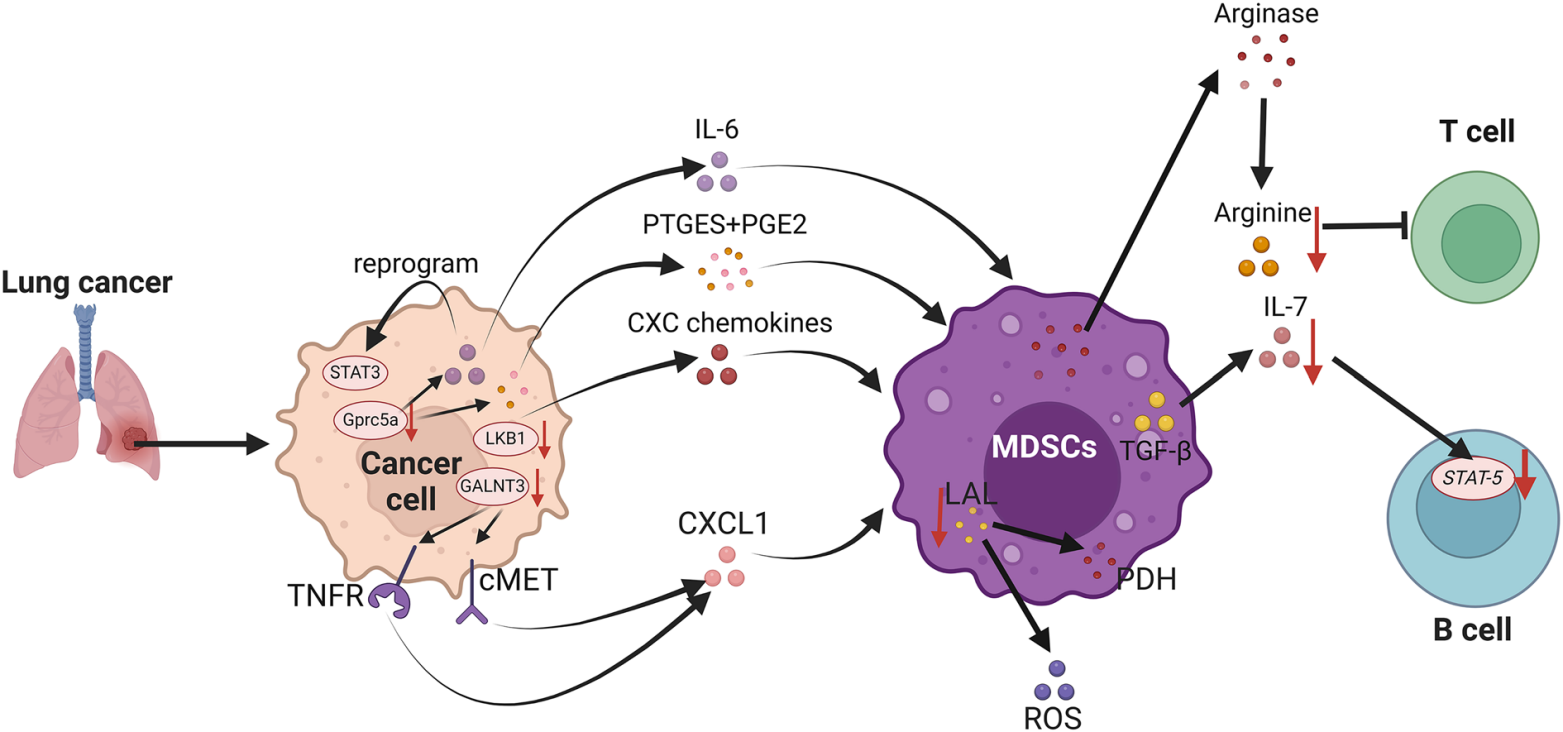
MDSCs in lung cancer. MDSCs inhibit the function and differentiation of T and B cells by depleting arginine and IL-7 in lung cancer. In addition, stimulated by the overexpression of the cytokines IL-6, PTGES, PGE2 and CXC in lung cancer cells, MDSCs are recruited to the TME, where they help tumors escape immune surveillance. Meanwhile, both PDH and ROS upregulation caused by LAL reduction in MDSCs suppressed immune function

Lung cancer (LC) is the second most common type of cancer after BC and is a global cancer burden, representing 11.4% of all cases and the main contributor of cancer deaths [121]. Although blocking the activation of "immune checkpoints" has shown therapeutic effects in most lung cancer patients, many of patients still do not benefit from this treatment. Over the past few years, many studies have reported the mechanisms by which MDSCs affect the LC tumor microenvironment (Fig. 5). According to multiplex quantitative immunofluorescence staining of non-small cell lung cancer (NSCLC) tissues, the proportion of CD11b + /HLA-DR − MDSCs-like cells was found to be dramatically more abundant in the tumor than in the matched non-tumor lung tissue, and increased expression of CD11b or HLA-DR was linked to a trend toward shorter 5-year survival [21].

In NSCLC, MDSCs further suppressed T-cell activity by increasing arginase expression and depleting arginine. In contrast, arginase inhibition caused tumor shrinkage by improving arginine levels and restoring T-cell function in mouse tumor models [146]. Furthermore, MDSCs directly affect B-cell differentiation and function through TGF-β-mediated Interleukin-7 (IL-7) deficiency and reduce downstream STAT-5 signaling [147]. Activation of STAT3 inhibits myeloid apoptosis, hinders cell differentiation and drives MDSCs expansion in cancer [148]. In CCSP-rtTA/(tetO)7-Stat3C bitransgenic mice, constitutively active Stat3C overexpression and sustained activation of the Stat3 signaling pathway induce spontaneous bronchoalveolar adenocarcinoma [149]. G-protein–coupled receptor family C member 5A (Gprc5a), a retinoic acid-inducible gene, is predominantly expressed in lung tissues [150]. Gprc5a-knockout (ko) mice is susceptible to developing spontaneous and carcinogen-induced lung cancer. However, the development of lung tumors in Gprc5a-ko mice is related to chronic inflammation [151]. In Gprc5a-ko mice, overexpression of IL-6 reprogrammed the STAT3 pathway and induced recruitment of PMN-MDSCs and polarized macrophages to evade host immunity, leading to metastasis of tumor cells within the mouse lung [152]. Furthermore, in spontaneously forming lung adenocarcinomas in Gprc5a-ko mice, membrane-bound PGE synthase (PTGES) and prostaglandin E2 (PGE2) overexpression induced the expression of cytokines and chemokines, such as G-CSF, GM-CSF, and TNF-α, which further induced the recruitment of PMN-MDSCs into the TME [153].

Recent studies have shown that within lung adenocarcinomas, the fungus Aspergillus sydowii, despite its low biomass, plays an important role in stimulating the immunosuppressive TME, thereby promoting lung tumor progression and is associated with poor patient prognosis. This is mainly due to the fact that Aspergillus sydowii, which is enriched within lung adenocarcinomas, can also secrete IL-1β via the β-glucan/Dectin-1/Caspase-recruitment domain 9 pathway, which mediates the recruitment and activation of MDSCs, especially PMN-MDSCs, and promotes lung cancer progression [154]. LKB1 inactivating mutations were found in approximately 20% of NSCLC patients and in 1/3 of KRAS-mutant NSCLC patients [155]. It has been reported that the absence of LKB1 in NSCLC is linked to immune-related features such as reduced neutrophil abundance and T-cell infiltration in the TME [156]. Additionally, in the LKB1-deficient mouse model of NSCLC, PMN-MDSCs were increased locally in the tumor microenvironment as well as systemically in the peripheral blood and spleen because of the increased secretion of C-X-C motif (CXC) chemokines with NH2-terminal Glu-Leu-Arg motifs in premalignant and cancer cells [157]. In addition, abnormal upregulation of the apoptosis inhibitor 6 in lung alveolar type II epithelial cells of mice promoted CD11bLy6G myeloid cell expansion in the lung and blood, leading to suppression of T-cell function and promoting the development of adenocarcinoma [158].

It has been reported that the level of polypeptide N-acetyl-galactosaminyltransferase 3 (GALNT3) expression is lower in LC tissue than in normal lung tissue and is correlated with poor prognosis in cancer patients [159]. This is because the recruitment of PMN-MDSCs can be impeded by GALNT3, which blocks the self-renewal of LC cells and leads to the downregulation of CXCL1 by decreasing the level of β-catenin, the nuclear localization of NF-κB and the c-MET-induced phosphorylation of AKT [160]. In human patients with NSCLC, the expression of LAL was markedly reduced in MDSCs, with significant expansion of these MDSCs subsets and upregulation of pyruvate dehydrogenase (PDH) in MDSCs [161]. In contrast, blockade of PDH in glycolysis reversed the immunosuppressive and tumor growth-stimulating effects of Lal MDSCs and reduced ROS overproduction [161]. CAFs are activated fibroblasts that have been shown to accelerate tumor growth and mediate tumor resistance to chemotherapy [162]. Induced by lung squamous cell carcinoma-derived CAFs, M-MDSCs can be functionally reprogrammed from monocytes, thereby inhibiting effector T-cell proliferation and IFN-γ production [78]. Therefore, MDSCs, which are immunosuppressive cells that inhibit the TME to attenuate the immune response, may be a new target for immune system therapy.

### Other cancers

In addition to the previously mentioned breast, lung, liver, prostate and melanoma cancers, MDSCs have been found to accumulate and participate in immune escape in a variety of other tumor types, such as pancreatic ductal adenocarcinoma (PDAC), colorectal cancer, glioblastoma multiforme (GBM), acute myeloid leukemia (AML), and sarcomas.

The levels of CD11bCD33CD15 MDSCs were increased in the peripheral blood, bone marrow and tumor tissue of patients with pancreatic adenocarcinoma [163]. Neutrophil-like MDSCs accumulate in the tumor tissue of patients with PDAC, and CD13hi neutrophil-like MDSCs exert immunosuppressive effects through ARG1 expression [164]. Significant Gr-1CD11b-induced MDSCs aggregation was found in the spleen, bone marrow and tumor tissue in a mouse model of pancreatic ductal adenocarcinoma [165]. PDAC cells produce cytokines involved in the induction, recruitment and stocking of MDSCs. This leads to the accumulation of MDSCs in tumors [166]. Moreover, CD200R expression is elevated on MDSCs in patients with PDAC, and interacted with CD200 in the TME to promote MDSCs expansion [167]. In contrast, intratumor accumulation of endogenous CD8 + T cells and apoptosis of tumor cells can be induced after targeting the depleted granulocytic MDSCs subpopulation in vivo [166]. For fatal malignancies such as PDAC, radiotherapy plays a crucial role in the treatment process [168]. However, radiation promotes the activation of MDSCs through increased lactate secretion, reprogramming the tumor microenvironment to a more immunosuppressive phenotype [169]. This contributes to the development of radioresistance in PDAC.

Research in mouse models of colitis-associated cancer (CAC) indicates that enhanced MDSCs accumulation and immunosuppression can drive tumorigenesis and progression during the long-term course of chronic inflammation [170]. As a sensor of bacterial-derived muramyl peptides, the Nod-like receptor protein Nod1 stimulates expansion and exerts immunosuppressive effects on M-MDSCs in colorectal cancer by expressing and maintaining ARG1 levels [171]. In addition, the PMN-MDSCs-derived exosome S100A9 promotes stemness in CAC cells in a HIF-1α-dependent manner [172]. In different mouse models of colorectal cancer, methyltransferase-like 3 promoted the expression of the basic helix-loop-helix family member e41 in a m6A-dependent manner and subsequently induced the transcription of CXCL1, which enhanced MDSCs migration in vitro via CXCR2 [173]. Chromosome X deletion in colorectal cancer increases tyrosine synthesis and secretion. Tyrosine is taken up by MDSCs and metabolized to homogentisic acid, which modifies the protein inhibitor of activated STAT3 via carbonylation of Cys 176 and alleviates the inhibitory function of the protein inhibitor of activated STAT3 on signal transducer and activator of transcription 5 transcriptional activity [174]. This promotes MDSCs survival and accumulation, allowing colorectal cancer cells to become invasive and metastatic [174]. Furthermore, Candida tropicalis in the gut enhances the immunosuppressive function of MDSCs by activating PKM2-dependent glycolysis, which in turn promotes colorectal cancer development [175].

In hematologic malignancies, MDSCs can also promote tumor progression and immunosuppression [176]. The frequency of CD14^+^ HLA-DR^low^ MDSCs was substantially increased in patients with confirmed AML, and effector T-cell function was inhibited in a manner dependent on IDO1 [177]. In AML, monocytes are prone to take up AML-derived extracellular vesicles (EVs) and subsequently differentiate, acquiring a CD14^+^HLA-DR^low^ phenotype and upregulating the expression of the immunoregulatory gene indoleamine-2,3-dioxygenase [178]. Furthermore, palmitoylation of proteins on the surface of AML-EVs activates Toll-like receptor 2 and thus triggers Akt/mTOR-dependent induction of M-MDSCs, making targeted protein palmitoylation a possible therapeutic target for improving the immune response in AML [178]. In addition, interleukin receptor-associated kinase 1 induces MDSCs through regulated IFN-γ signaling to promote immune escape in fibroblast growth factor receptor-1 (FGFR1)-driven hematologic malignancies [179].

CXCR2 ligands are produced in human pediatric sarcomas and are elevated in the serum when sarcomas metastasize [180]. A study showed that murine rhabdomyosarcoma induced the expansion of CXCR2CD11bLy6G^++hi^ MDSCs and that CXCR2-mediated recruitment of MDSCs into tumors exerted immunosuppressive effects. In the peripheral blood of patients with GBM, the levels of MDSCs, mainly CD15CD14 + neutrophils, substantially increase, and the density of MDSCs within the tumor increases with the progression of glioma, which is closely related to patient survival [181, 182]. Leukocyte immunoglobulin-like receptor subfamily B member 4 promotes the immunosuppressive function of M-MDSCs by regulating the M2 polarization of MDSCs and inhibiting the secretion of miR-1 family miRNAs, thus helping tumors evade immune surveillance [183].

## The main treatment for MDSCs

**Fig. 6:**
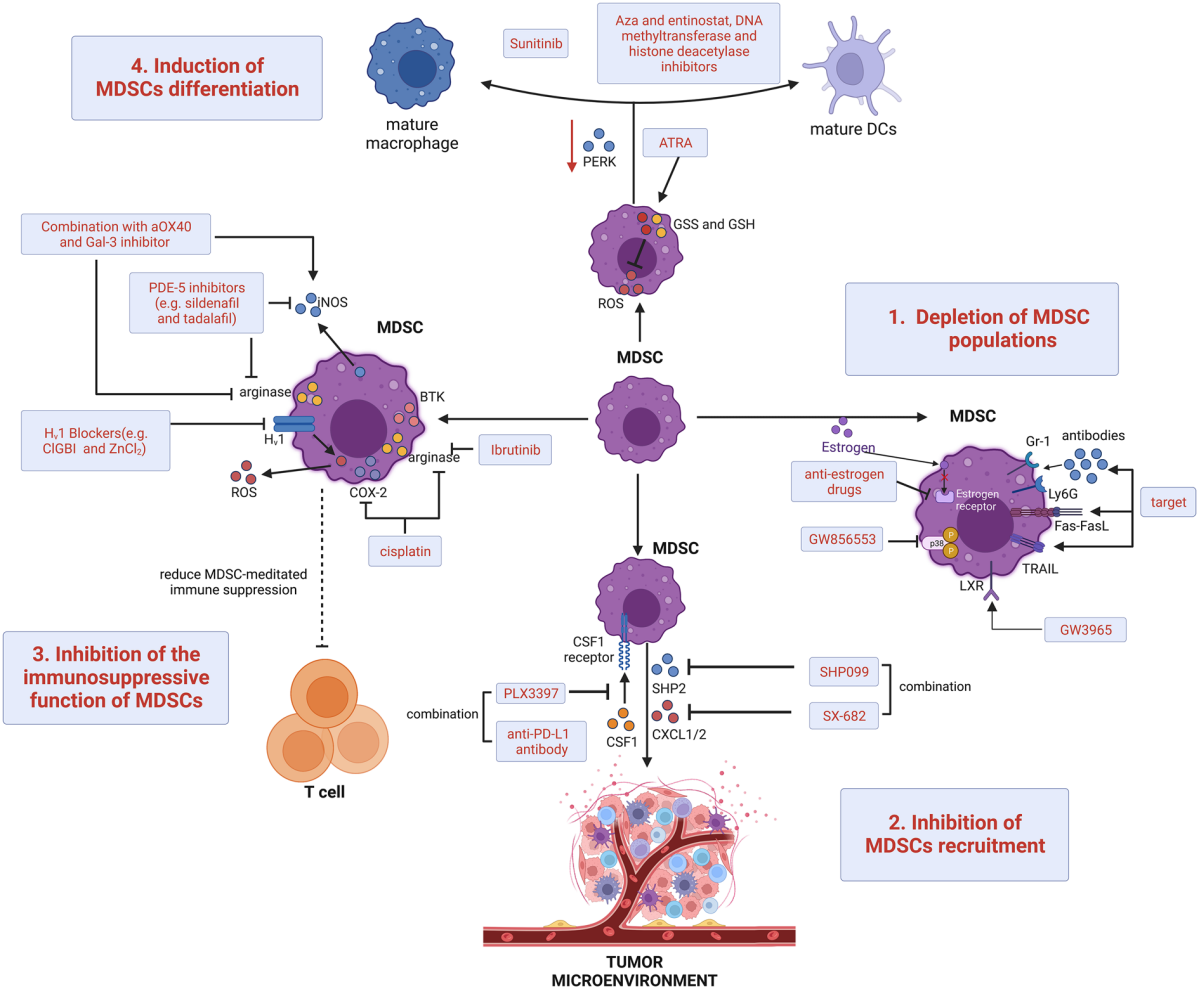
Primary therapies targeting MDSCs. MDSCs are eliminated, and/or their immunosuppressive activity is inhibited by different strategies, including (1) depletion of MDSCs populations, (2) inhibition of MDSCs recruitment, (3) inhibition of MDSCs immunosuppressive activity, and (4) induction of MDSCs differentiation

Due to their abundance and high immunosuppressive capacity, a large body of evidence suggests that tumor-associated myeloid cells have a profound impact on immunotherapy resistance [184]. In the last few years, an increasing number of preclinical studies and clinical trials have been conducted to validate the potential safety and benefits of inhibiting MDSCs alone or in conjunction with radiation, chemotherapy and immunotherapy for the treatment of cancer [185]. The main therapeutic strategies used in current studies to eliminate MDSCs and/or inhibit their immunosuppressive activity within the TME include (i) depletion of MDSCs populations; (ii) inhibition of MDSCs recruitment to tumor sites; (iii) attenuation of the inhibitory activity of MDSCs by targeting specific molecular pathways involved in MDSCs-mediated immune escape processes; and (iv) promotion of MDSCs differentiation, such as differentiation into M1 macrophages or dendritic cells (Fig. 6).

Intensive studies have explored new ways to target and deplete MDSCs. In mouse models, apoptosis in MDSCs can be mediated by targeting antibodies to the surface markers Gr-1 or Ly6G, inducing Fas-FasL or targeting the TNF-related apoptosis-induced ligand (TRAIL) receptor [186–188]. Similarly, in models of female malignancy estrogen and its receptor alpha signaling cause MDSCs amplification and enhanced immunosuppressive activity through altered pSTAT3 signaling, which supports the idea that more specific anti-estrogen drugs could complement emerging immunotherapies [189]. In addition, the use of the p38 MAPK inhibitor GW856553 in murine HCC models with cirrhosis effectively inhibited the enhancer reprogramming of M-MDSCs development and immunosuppression induced by activated hepatic stellate cells [190]. Furthermore, activation of the therapeutic liver-X nuclear receptor and its transcriptional target apolipoprotein E signaling by the application of the selective agonist GW3965 directly inhibited the survival of MDSCs in murine models and in patients treated in a first-in-human dose escalation phase 1 trial [191]. Some cytotoxic chemotherapies, such as carboplatin and paclitaxel, can also reduce the number of circulating MDSCs in tumor patients [192], some of which are now thought to support the antitumor effects of certain regimens. In addition, the combination of chemotherapy with other therapies to target MDSCs has demonstrated preclinical and clinical antitumor effects (Table 1).

**Table 1:** Clinical trials targeting MDSCs in tumors

Target	Intervention	Conditions	Phase	Status	Number Enrolled	NCT
Adenosine A2B Receptor antagonist; Chemotherapy	PBF-1129 and Nivolumab	NSCLC	I	Recruiting	30	NCT05234307
Chemotherapy; Electrothermal therapy	Gemcitabine; Focused Ultrasound; Gemcitabine and Focused Ultrasound	Breast Cancer	I	Recruiting	48	NCT04796220
Chemotherapy	Tadalafil	Astrocytoma	I	Completed	18	NCT04757662
Endocrine Therapy; Chemotherapy	Abemaciclib; Fulvestrant; Aromatase Inhibitors	Breast Cancer	II	Active, not recruiting	18	NCT04352777
Anti-PD-1; Chemotherapy	Nivolumab; Nivolumab and Gemcitabine	NSCLC	II	Terminated	3	NCT03302247
Chemotherapy	Fludarabine; Busulfan; Methotrexate	Leukemia	I	Active, not recruiting	20	NCT02916979
CXCR1/2 antagonist; Anti-PD-1	SX-682 and Pembrolizumab	Melanoma	I	Recruiting	77	NCT03161431
PDE-5 inhibitor	Tadalafil	Head and Neck Squamous Cell Carcinoma	II	Completed	40	NCT01697800
Anti-PD-1; ATRA	Pembrolizumab with ATRA	Melanoma	I/II	Active, not recruiting	26	NCT03200847
Anti-PD-1; ATRA	ATRA and Atezolizumab	NSCLC	I	Recruiting	18	NCT04919369
ATRA; Anti-CTLA-4	ATRA; Ipilimumab	Melanoma	II	Active, not recruiting	10	NCT02403778
H2 receptor antagonist	Ranitidine	Cancer	IV	Completed	30	NCT03145012
TLR9 agonist; Anti-PD-1	CMP-001 and Nivolumab	Melanoma; Lymph Node Cancer	II	Active, not recruiting	34	NCT03618641
Chemotherapy; Anti-VEGF	Capecitabine; Bevacizumab	Recurrent Glioblastoma	I	Active, not recruiting	12	NCT02669173

MDSCs, myeloid-derived suppressor cells; MNPs, mononuclear phagocytes; DCs, dendritic cells; ImCs, immature cells; MSCs, myeloid suppressor cells; PMN-MDSCs, polymorphonuclear-MDSCs; M-MDSCs, monocytic MDSCs; ARG1, arginase 1; MMP-9, metalloproteinase-9; PD-L1, programmed death ligand 1; HLA-I, human leukocyte antigens class I; CMPs, common myeloid progenitors; GMPs, granulocyte–macrophage progenitors; MB, myeloblasts; MDP, monocyte/macrophages and dendritic cell; IL-17A, Interleukin-17A; GM-CSF, granulocyte–macrophage colony-stimulating factor; G-CSF, granulocyte colony-stimulating factor; TNF-α, tumor necrosis factor-alpha; CCR2, C–C chemokine receptor 2; CCR5, C–C chemokine receptor 5; TGF-β, transforming growth factor beta; CD62L, L-selectin2; ADAM17, a disintegrin and metalloproteinase domain 17; TACE, TNF-α-converting enzyme; HMGB1, high mobility group box-1; ROS, reactive oxygen species; VEGF, vascular endothelial growth factor; TCR, T-cell receptor; MHC, major histocompatibility complex; RNS, reactive nitrogen species; NO, nitric oxide; Trp, tryptophan; TME, tumor microenvironment; CAT-2B, cationic amino acid transporter protein; IDO1, indoleamine-2,3-dioxygenase 1; AhR, aryl hydrocarbon receptor; PD-1, programmed cell death 1; VISTA, V-domain Ig suppressor of T-cell activation; Gal-9, galactose lectin-9; AML, acute myeloid leukemia; TIM-3, T-cell immunoglobulin and mucin structural domain 3; STING, stimulator of interferon genes; TIGIT, T-cell immunoglobulin and ITIM domain; HNSCC, head and neck squamous cell carcinoma; NK, Natural killer; NKG2D, natural killer group 2D; IFN-γ, interferon-γ; STAT3, signal transducer and activator of transcription 3; NF-Κb, nuclear factor-κB; IL-10, interleukin-10; IL-12, interleukin-12; Treg, regulatory T; LTB4, leukotriene B4; EV, extracellular vesicles; HSP90α, heat shock protein 90α; NLRP3, NOD-like receptor protein 3; CCN4, Cell Communication Network Factor 4; HCC, Hepatocellular carcinoma; CAFs, cancer-associated fibroblasts; MIF, migration inhibitory factor; SLC7A11, solute carrier family 7 member 11; CSF1, colony-stimulating factor 1; SLC7A2, Solute carrier family 7 member 2; PI3K, phosphatidylinositol 3-kinase; AKT, protein kinase B; RIP3, receptor-interacting protein kinase 3; ENTPD2, ectonucleoside triphosphate diphosphohydrolase 2; HIF-1, hypoxia-inducible factor-1; ATP, adenosine triphosphate; 5'-AMP, 5'-Adenosine monophosphate; PIWIL1, Piwi Like RNA-Mediated Gene Silencing 1; FAO, fatty acid β-oxidation; AR, androgen; CCRK, cell cycle-related kinase; EZH2, Enhancer of zeste homolog 2; BC, breast cancer; GMPs, granulocyte-monocyte progenitors; GPs, granulocyte progenitors; BM, bone marrow; CCL20, C–C motif chemokine ligand 20; BCSC, breast cancer stem cells; NO, nitric oxide; CEBPB, CCAAT/enhancer-binding protein beta; AMPK, AMP-activated protein kinase; MAPK, mitogen-activated protein kinase; AMPKα, AMP-activated protein kinase alpha; eMDSCs, early myeloid-derived suppressor cells; JAK, Janus kinase; SOCS3, suppressor of cytokine signaling-3; PIAS3, protein inhibitor of activated STAT-3; SMAD3, Smad family member 3; KAT6A, lysine acetyltransferase 6A; TRIM, tripartite motif‐containing; Enpp1, ectonucleotide pyrophosphatase/phosphodiesterase 1; NET, neutrophil extracellular traps; PCa, Prostate cancer; ICB, immune checkpoint blockade; CRPC, castration resistant prostate cancer; IL-23, Interleukin-23; CHD1, chromodomain-helicase-DNA-binding protein 1; PTEN, Phosphatase and tensin homolog; LC, Lung cancer; NSCLC, non-small cell lung cancer; IL-7, Interleukin-7; Gprc5a, G-protein–coupled receptor, family C, member 5A; PTGES, PGE synthase; PGE2, prostaglandin E2; GALNT3, polypeptide N-acetyl-galactosaminyltransferase 3; PDH, pyruvate dehydrogenase; CAFs, Cancer-associated fibroblasts; PDAC, pancreatic ductal adenocarcinoma; AML, acute myeloid leukemia; CAC, colitis-associated cancer; FGFR1, fibroblast growth factor receptor-1; TRAIL, TNF-related apoptosis-induced ligand; SHP2, phosphatase 2; Aza, azacytidine; PDE-5, Phosphodiesterase-5; CIK, cytokine-induced killer; Gal-3, alactose lectin-3; Hv1, voltage-gated proton channels; COX-2, cyclo-oxygenase 2; BTK, Bruton’s tyrosine kinase; UPR, unfolded protein response;ATRA, All-trans retinoic acid

Reducing the recruitment of MDSCs to tumor sites is one of the main therapeutic approaches to re-establish the immune microenvironment and improve the success of immunotherapy. Blocking chemokines and their interactions with ligands is an effective target for reducing the transport of MDSCs [193]. Src homology-2-containing protein tyrosine phosphatase 2 (SHP2) inhibitors (e.g., SHP099) have antitumor effects on Models with KRAS-mutant and EGFR-mutant NSCLC [194, 195]. However, both SHP2 inhibitors and other RAS/ERK pathway inhibitors cause the recruitment of MDSCs by inducing NF-kB-dependent CXCR2 ligand production [196]. Therefore, SHP2 inhibitors need to be combined with CXCR1/2 (e.g., SX682) inhibitors and improve survival in multiple NSCLC models. Adjuvant epigenetic treatment with low-dose DNA methyltransferase and the histone deacetylase inhibitors entinostat and 5-azacytidine (Aza) after primary tumor resection within mouse models inhibited tumor cell dissemination by reducing the transport of MDSCs by downregulating CCR2 and CXCR2 and by encouraging MDSCs differentiation [197]. PMN-MDSCs transport was significantly inhibited in mouse tumor models following the application of SX-682 (CXCR1 and CXCR2 inhibitor), which enhanced the ability to respond to programed death-axis ICB and adoptive transfer of engineered T cells [198]. The CSF1/CSF1 receptor (CSF1R) pathway is another clear target for reducing MDSCs recruitment. CSF1/CSF1R-targeted drugs have been studied in a variety of tumor types [199–201]. In combination with anti-PD-L1 blockers in a mouse model of HCC, CSF1R inhibitors (e.g., PLX3397) significantly inhibited the recruitment of MDSCs, TAM infiltration and M2 polarization, leading to reversal of the immunosuppressed state of the HCC microenvironment [202]. In addition, the combined application of CSF1R and CXCR2 inhibitors in multiple mouse tumor models significantly reduced the recruitment of TAMs and PMN-MDSCs to the tumor site and significantly reduced tumor growth [203]. Furthermore, inhibition of CCR2 with PF-04136309 or RS504393 blocked the recruitment of macrophages and M-MDSCs in a mouse model of pancreatic cancer. However, this leads to an increase in the number of neutrophils and PMN-MDSCs within the tumor. Combining PF-04136309 with the CXCR2 inhibitor SB225002 or the CXCL8 neutralizing antibody was able to further increase chemotherapeutic efficacy [204]. This suggests the possibility of some functional compensation between M-MDSCs and PMN-MDSCs. Blockade of the MDSCs-secreted factor prokineticin (Bv8) can also inhibit the transport of MDSCs to tumors through anti-angiogenic effects in a mouse model of pancreatic cancer [205]. To target MDSCs recruitment, SX-682 is currently being used in connection with pembrolizumab in a phase I trial for the treatment of melanoma (NCT03161431).

Inhibition of the immunosuppressive effect of MDSCs is another therapeutic strategy to target MDSCs. Phosphodiesterase-5 (PDE-5) inhibitors can suppress the function of MDSCs by reducing the levels of iNOS and arginase. In mouse models, PDE-5 inhibitors (e.g., sildenafil and tadalafil) activate antitumor immunity and prolong the survival of tumor-bearing mice [206, 207]. In mouses model of hepatocellular carcinoma, systemic treatment with PDE-5 inhibitors may also prevent the accumulation of MDSCs in the TME induced by cytokine-induced killer (CIK) cell-derived immunotherapy in HCC through ARG1 and iNOS blockade, increasing the antitumor function of CIK cell therapy [208]. Blockade of Bv8 inhibits the immunosuppressive properties of MDSCs by inducing increased expression of IDO, ROS1 and iNOS in a mouse breast cancer model [209]. In the tumor-bearing mouse models, the extracellular generation of ROS is one of the mechanisms by which MDSCs exert their immunosuppressive function. Combination treatment with an agonist anti-OX40 antibody (aOX40) and a galactose lectin-3 (Gal-3) inhibitor induces a decrease in ARG1 and an increase in iNOS and reduces M-MDSCs-mediated immune suppression, thereby increasing CD8 + T-cell recruitment [210]. In mouse models, inhibition of voltage-gated proton channels (Hv1) by 5-chloro-2-guanidinobenzimidazole or ZnCl2 and its consequent pH reduction inhibit NADPH oxidase 2-mediated ROS production, thereby reducing the immunosuppressive effects of MDSCs [211]. Ibrutinib reduces monocyte and granulocyte MDSCs-mediated NO production, mRNA expression of immunosuppressive cytokines and T-cell suppression in a mouse model of neuroblastoma through inhibition of Bruton’s tyrosine kinase (BTK) [212]. In order to inhibit the immunosuppressive activity of MDSCs, the clinical studies have reported fewer circulating MDSCs, lower expression of arginase and iNOS in these cells, and higher levels of tumor-specific T cells in patients with head and neck cancer and multiple myeloma treated with the PDE-5 inhibitor tadalafil [213, 214]. In addition, tadalafil (PDE-5 inhibitor) has been clinically tested in patients with head and neck cancer in a phase II trial (NCT01697800). In patients with HNSCC and melanoma treated with cisplatin, the expression of ARG1 and cyclo-oxygenase 2 (COX-2) was significantly reduced in M-MDSCs, and the ability of M-MDSCs to block activated T-cell responses in isolation was markedly reduced [215]. These findings suggested that platinum-based chemotherapeutic agents may enhance the efficacy of immunotherapy by overcoming M-MDSCs-mediated immunosuppression.

A final therapeutic strategy to re-establish T-cell activity and immunotherapeutic success is the induction of MDSCs to differentiate into mature, non-suppressive myeloid cells. Although moderate activation of unfolded protein response (UPR)-related signaling helps immune cells differentiate and function physiologically, persistent and maladaptive initiation of UPR drivers facilitates immunodeficiency [216, 217]. Increased PKR-like endoplasmic reticulum kinase signaling is characteristic of the UPR in MDSCs of a tumor-bearing mouse model, whose deletion converts MDSCs into cells that activate CD8 + T-cell immunity in tumor beds [218]. All-trans retinoic acid (ATRA), a derivative of vitamin A, has been found to be highly active against MDSCs [219]. In vivo administration of ATRA stimulates MDSCs from tumor-bearing mice to differentiate into mature myeloid cells [220]. ATRA-induced MDSCs differentiation involves ATRA specifically upregulating the expression of glutathione synthase and glutathione in MDSCs, thereby neutralizing ROS and driving myeloid differentiation [221]. In preclinical breast cancer models, ATRA was found to enhance the efficacy of antiangiogenic therapy for breast cancer by depleting MDSCs [222]. In addition, by promoting the expression of differentiation genes in peritoneal PMN-MDSCs, the receptor tyrosine kinase inhibitor sunitinib promotes the differentiation of MDSCs into mature polynuclear MDSCs in a mouse model of endometriosis [223]. Finally, adjuvant epigenetic therapy with low-dose Aza, entinostat, DNA methyltransferase and histone deacetylase inhibitors within mouse models was used to inhibit tumor cell dissemination by disrupting the premetastatic microenvironment through the promotion of MDSCs differentiation to a more mesenchymal macrophage-like phenotype [197]. To induce differentiation of MDSCs, high plasma concentrations (> 150 ng/mL) of ATRA in patients with metastatic renal cell carcinoma promote differentiation of MDSCs to APC precursors, thereby eliminating MDSCs-mediated immunosuppression [224]. Currently, combinations of ATRA and ICIs is used in several clinical trials, including pembrolizumab, atezolizumab, and ipilimumab (NCT03200847, NCT04919369, and NCT02403778, respectively).

## Conclusion and prospects

In recent years, significant progress has been made in cancer immunotherapy, especially in the treatment of various types of solid tumors (e.g., melanoma, breast cancer, and non-small cell lung cancer). Nevertheless, there are still many patients who do not benefit due to drug resistance or relapse, which is most likely attributable to multiple immunosuppressive cells in the TME. As prognostic and predictive biomarkers, MDSCs play an essential role in development of tumor immune escapes. Patients may benefit from targeting MDSCs due to the diverse roles of MDSCs in TME. This review describes some of the mechanisms by which MDSCs participate in tumor immune escape through immunosuppression and summarizes the specific pathways by which MDSCs have been involved in various types of tumor immune escape in recent years. Unlike Treg cells or checkpoint molecules, MDSCs do not seem to exist in a steady state. This provides a unique opportunity to target MDSCs with potentially no side effects. However, current therapeutic strategies targeting MDSCs are only partially effective [225]. First, MDSCs are highly heterogeneous in different cancers, and identification of human MDSCs phenotypes is a challenge. Second, majority of studies in humans have focused only on circulating MDSCs, and very little is known regarding tumor-infiltrating MDSCs. Then, the complex nature of the TME and the multifunctional nature of MDSCs, where inhibitory mechanisms of MDSCs are unlikely to function simultaneously, make it difficult to identify the primary targets against MDSCs. Finally, targeting M-MDSCs leads to an increase in PMN-MDSCs and vice versa, so that targeting one type of MDSCs alone may not be effective. Therefore, it seems impossible to control or eliminate MDSCs via a single approach and thus induce a significant antitumor effect, and the combination of MDSCs-targeted therapy with other anticancer therapies should be the preferred strategy. In addition, there is still a need to further investigate the major mechanisms and upstream signals underlying the emergence, amplification and immunosuppressive functions of MDSCs in various tumors. Advances in this area should help rationalize the design of new strategies against MDSCs to enhance the clinical response to current immunotherapies and improve patient prognosis. Future studies should clarify how much efficacy and survival benefit combination therapies can provide to cancer patients. A large-scale clinical trial and in-depth preclinical study are needed to confirm these questions.

## Data Availability

Not applicable.
